# Simulation Study on Jet Formability and Damage Characteristics of a Low-Density Material Liner

**DOI:** 10.3390/ma11010072

**Published:** 2018-01-04

**Authors:** Liangliang Ding, Wenhui Tang, Xianwen Ran

**Affiliations:** College of Science, National University of Defense Technology, Changsha 410073, China; dingliangliang14@nudt.edu.cn (L.D.); ranxianwen@nudt.edu.cn (X.R.)

**Keywords:** explosive reactive armor, low-density material, shaped jet, liner, tandem warhead

## Abstract

The shaped charge tandem warhead is an effective weapon against the ERA (explosive reactive armor). Whether the pre-warhead can reliably initiate the ERA directly determines the entire performance of the tandem warhead. The existing shaped charge pre-warhead mostly adopts a metal shaped jet, which effectively initiates the ERA, but interferes the main shaped jet. This article, on the other hand, explores the possibility of producing a pre-warhead using a low-density material as the liner. The nonlinear dynamic analysis software Autodyn-2D is used to simulate and compare three kinds of low-density shaped jets, including floatglass, Lucite, and Plexiglas, to the copper shaped jet in the effectiveness of impacting ERA. Based on the integrative criteria (including *u*-*d* initiation criterion, explosive reactive degree, explosive pressure, and particle velocity of the panels), it can be determined whether the low-density shaped jet can reliably initiate the sandwich charge. The results show that the three kinds of low-density shaped jets can not only initiate the reaction armor, but are also superior to the existing copper shaped jet in ductility, jet tip velocity, jet tip diameter, and the mass; namely, it is feasible to use the low-density material shaped jet to destroy the ERA.

## 1. Introduction

Local wars in recent years have shown that the modern armored targets, such as tanks and armored vehicles, are still the main combat systems on the ground battlefield, which integrates firepower, protection, and mobility. The shaped charge warhead is one of the most important anti-armor ammunition, and a great deal of manpower, material, and financial resources have been spent on improving its power and performance. In 1969, Held found that the sandwich structure (metal panel/explosive/metal panel) can significantly reduce the penetration ability of a shaped jet and kinetic rod, then applied for a patent in 1970, which then became the prototype of ERA (explosive reactive armor). The ERA is a revolution in the field of armor protection for its wide use in main battle tanks and armored vehicles, to name a few: Russia′s T-90 main battle tank, Israel′s MK4 main battle tank, the United States M1A1/M1A2 main battle tank, China′s 99 series main battle tank and Germany′s Leopard II main battle tank.

**Figure 1:**
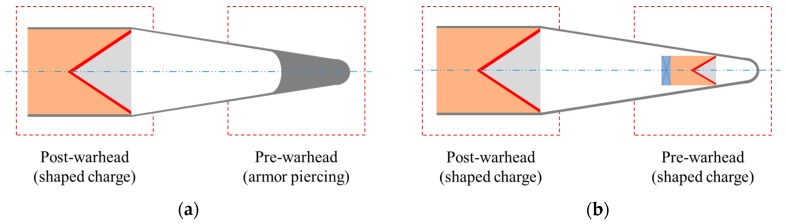
(**a**) Schematic diagram of the armor piercing-shaped charge type tandem warhead; and (**b**) schematic diagram of the shaped charge-shaped charge type tandem warhead.

The wide application of ERA has posed a great threat to the traditional anti-armor ammunition. When equipped with ERA, the armored target′s protection ability, battlefield survival ability, and the sustained combat ability are greatly enhanced. The experimental results show that the protective efficiency of the single-layer ERA structure to the conventional shaped charge warhead is 70–90% [1,2]. Therefore, in order to effectively deal with ERA and eliminate the interference of the ERA action field on the shaped jet, the concept of a shaped charge tandem warhead was put forward. At present, the shaped charge tandem warhead that is widely used mainly includes two kinds of structures; it is either the armor piercing-shaped charge type or the shaped charge-shaped charge type. The armor piercing-shaped charge type tandem warhead is mainly composed of a pre-warhead (armor piercing or EFP) and post-warhead (shaped charge), as shown in Figure 1a. Its working principle is that the pre-projectile penetrates and passes through the ERA at a certain speed without causing the sandwiched charge to explode, and then the main charge is initiated after a certain delay and forms the shaped jet to impact the ERA through the hole formed by the pre-impact; thus, the ERA loses the ability to interfere with the shaped jet. The shaped charge-shaped charge type tandem warhead is mainly composed of a pre-warhead (shaped charge) and post-warhead (shaped charge), as shown in Figure 1b. Its working principle is that the pre-charge is initiated first and forms a shaped jet to initiate the sandwich charge placed in the middle of ERA, and after a certain delay the main shaped jet formed by the post-charge can penetrate the ERA without interference [3].

Held [4,5,6,7,8,9,10,11] studied the interaction mechanism between the armor and the shaped jet through a large number of experiments, and obtained the interference law of sandwich charge thickness, detonation velocity, metal plate thickness, and jet angle on the shaped jet. Mayseless [12] conducted experiments to study the interaction between the shaped jet and the metal panel, and proposed the pebble interference model, in which the metal panel generates periodic interference to the shaped jet. In the subsequent study, Mayseless [13] also found that the jet diameter decreases after the jet passes through the ERA, and then established a continuous interference model (Grazing model). Koch [14] studied the change of the initiation criterion values of ERA with the variation of the shaped jet penetration normal through experiments. Helte [15] carried out a series of experiments on the penetration of ERA by the shaped jet of the pre-warhead, and found that the shaped jet formed by the alumina powder, aluminum powder, and glass material liner can perforate the ERA without causing explosion.

Through the above literature research, it can be found that the reaction mechanism and initiation criterion of the shaped jet to the ERA have been relatively well studied, and metal shaped jet was used to damage ERA in most of the previous work. In the future development of weapons, the warhead mass, economic cost, initiation safety, and other performance indicators will become more important factors. Therefore, this article takes the shaped charge-shaped charge type tandem warhead as the research prototype and hopes to explore the application of low-density materials in the shaped charge warhead based on the damage effectiveness of a low-density shaped jet on ERA targets.

## 2. Initiation Criteria of ERA

To initiate the ERA by shaped jet is actually to initiate the charge in between the sandwich structure (front panel/sandwich charge/rear panel). Whether the sandwiched charge can be initiated by a shaped jet is affected by the following factors: the jet velocity, jet diameter, thickness of the sandwich charge, thickness of panels, and the material of the shaped jet [16].

In the interaction between shaped jet and sandwich charge, the jet velocity determines the magnitude of the incident impact force. The higher the jet velocity, the higher the pressure generated by the impact and the stronger the excitation reaction. Therefore, jet velocity is the main control parameter in the process of initiating ERA, which has been reflected in the critical criteria for the detonation of explosives. The jet diameter mainly influences the jet precursor wave; since the jet precursor wave is generally curved, the greater the jet diameter, the smaller the curvature of the precursor wave, and less easily is the precursor wave zone affected by lateral rarefaction waves. At the same time, the jet penetrates into the ERA with high temperature, high pressure, and high strain rate, and the influence of the jet density on the penetration process is also very important, which mainly affects the penetration depth. In addition, with the increase of thickness and density of the sandwich charge, it is more difficult to initiate.

According to the above analysis, many scholars have studied the mechanism and process of the anti-ERA, the initiation criteria and threshold under many specific conditions were obtained, and many mature empirical formulas and critical conditions were established. Several widely used initiation criteria listed below.

### 2.1. p-τ Initiation Criterion

Walker and Wasley [17] proposed a one-dimensional short pulse initiation criterion in 1969, which was expressed as the critical energy on unit area. The expression of critical energy *E_cr_* is as follows:

(1) $$E_{cr}=p^{2}\tau /\rho_{0}U$$

where *p* is the initial shock wave pressure, *τ* is the duration of the pressure *p* in the explosive, *U* is the shock wave velocity, and *ρ*_0_ is the explosive density.

According to the critical energy theory of the impact initiation of heterogeneous explosives, when *p*^2^*τ* exceeds the critical explosive initiation threshold, it can arouse the detonation of explosives. Thus, the relationship between the critical initiation energy *E_c_* and *p*^2^*τ* is as follows:

(2) $$E_{c}=p^{2}\tau =const$$

### 2.2. v-d Initiation Criterion

In general, the loading area is very small under the impact of a projectile, fragments, or shaped jet. According to Held′s study [18], the influence of the pressure pulse width *τ* can be ignored when the aspect ratio of projectile is greater than 1/5. Held proposed an initiation criterion for high energy explosives by the shaped jet in two-dimensional loading case:

(3) $$K=v^{2}d$$

where *K* is the critical initiation threshold of explosives, *v* is the tip velocity of shaped jet, and *d* is the diameter of shaped jet. In engineering applications, if the cross-section of the penetrator is not circular, so the criterion is revised as follows:

(4) $$K=v^{2}\sqrt{A}$$

where *A* is the cross-sectional area of the penetrating body.

### 2.3. u-d Initiation Criterion

In the follow-up research, it was found that the jet density has a certain influence on the threshold velocity. Based on the numerical calculation, Mader [19,20] used a linear function to fit and revise the *v*-*d* initiation criterion:

(5) $$K=\rho_{p}v^{2}d$$

At the same time, Chick and Hatt [21] fit and revised the *v*-*d* initiation criterion based on experimental results of steel and aluminum jet:

(6) $$K=\sqrt{\rho_{j}}v^{2}d$$

It is clear that the two revised criteria above present significance in the initiation thresholds for the same explosives. Through comparative analysis, Held [22,23] concluded the general rule that the stagnation point pressure determined the difficulty of initiating the high explosive, and he revised the *v*-*d* initiation criterion as follows:

(7) $$K=u^{2}d$$

The above equation is the *u*-*d* initiation criterion, where u is the penetration velocity of the jet. In the hydrodynamic process, the penetration velocity *u* can be given by the Bernoulli equation:

(8) $$u=v/\left(1+\sqrt{\rho_{\mathrm{HE}}/\rho_{j}}\right)$$

where *v* is the tip velocity of shaped jet, *d* is the diameter of shaped jet, $\rho_{\mathrm{HE}}$ is the density of high explosive charge, and $\rho_{j}$ is the density of the shaped jet.

## 3. Simulation

The numerical simulations were carried out using Autodyn-2D (Century Dynamics, Fort Worth, TX, USA), which is an interactive non-linear explicit dynamics analysis software, widely used in simulating detonation, impact, armor-piercing, ballistics, and other problems. The software includes its own material library, and provides different state equations, strength models and failure and erosion models.

### 3.1. FE Model

**Figure 2:**
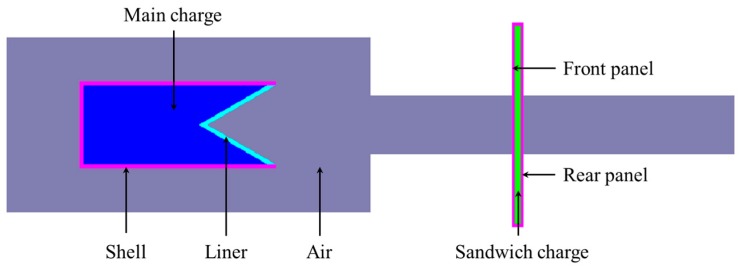
Finite element model.

The entire model mainly consists of seven parts, namely, air, main charge, shell, liner, sandwiched charge, front panel, and rear panel. As the entire finite element model has the characteristics of axial symmetry, the two-dimensional axisymmetric model is used in the simulation for computational efficiency. The finite element model is shown in Figure 2.

The explosion of the shaped charge and the collapse of the liner involve great deformation, so the fluid-solid coupling method was used in this article. The entire model was divided into two parts, he Lagrange part and Euler part. The main charge, air, liner, and sandwich charge were meshed as the Euler part to deal with great deformation, while the shell, front panel, and rear panel were meshed as the Lagrange part for fracture and fragmentation. To ensure the accuracy of the numerical simulation, the air domain adopts the center region encrypted gradient mesh. The flow-out boundary was set for the Euler boundary to eliminate the influence of the boundary effect. The contact between the Lagrange mesh and the Euler mesh was defined as automatic, and the initiation mode was center point initiation. In addition, the unit system of FE model was chosen as cm-g-μs.

The geometrical parameters of the entire model are shown in Table 1. A series of Gauss points were set in the model to facilitate the analysis of simulation results, and the specific numbers and coordinates of these Gauss points are shown in Table 2.

### 3.2. Modeling Material

Here, the main charge material was chosen as compB explosive, the sandwich charge as compBJJ1 explosive, the liner as copper, floatglass, Lucite, and Plexiglas, and the shell and panel as steel_1006. Each part of the corresponding material parameters and material model were taken from the Autodyn database, the material models and related parameters used in each section will be described below.

#### 3.2.1. Material Properties of Air

The equation of state of air was chosen as Ideal Gas. The model needs to be given an initial temperature of 15 °C and an initial internal energy of 2.068 × 10^5^ kJ∙kg^−1^.

(9) P=ρ(γ−1)E

(10) $$\gamma =C_{p}/C_{v}$$

(11) $$E=C_{v}T$$

where *P*, *ρ*, and *γ* are the pressure, density, and polytropic index of the gas, respectively, *C_p_* and *C_v_* are the specific heat at constant pressure and specific heat at constant volume, *T* is the temperature, and *E* is the internal energy of gas. The specific material parameters are shown in Table 3.

#### 3.2.2. Material Properties of the Main Charge

The Jones-Wilkins-Lee equation of state (EOS_JWL) was chosen to describe the material properties of the compB explosive. The EOS_JWL can accurately describe the volume, pressure, and energy characteristics of gas products in the process of detonation, which is expressed as follows:

(12) $$P=A\left(1-\frac{\omega }{R_{1}V}\right)e^{-R_{1}V}+B\left(1-\frac{\omega }{R_{2}V}\right)e^{-R_{2}V}+\frac{\omega E_{0}}{V}$$

where *A*, *B*, *R*_1_, *R*_2_, *ω* are material constants, *V* is the initial relative volume, *E*_0_ is the initial specific internal energy, and *P* is the detonation pressure. The specific parameters are listed in Table 4.

#### 3.2.3. Material Properties of the Sandwich Charge

The shock initiation process of heterogeneous explosives is divided into three stages, namely, ignition, growth, and completion. At present, the numerical model that can describe the behavior and characteristics of the impact initiation process is the Lee-Tarver ignition growth model. In the Lee-Tarver model, the state of explosive may be completely reactive, unreacted, or both coexist. Therefore, the Lee-Tarver model was selected to characterize the impact initiation process of compBJJ1 explosives. The reaction rate equation of the model is as follows:

(13) $$\frac{d\lambda }{dt}=I{\left(1-\lambda \right)}^{b}{\left(\frac{\rho }{\rho_{0}}-1-a\right)}^{x}+G_{1}{\left(1-\lambda \right)}^{c}\lambda^{d}p^{y}+G_{2}{\left(1-\lambda \right)}^{e}\lambda^{g}p^{z}$$

where *I*, *G*_1_, *G*_2_, *a*, *b*, *c*, *d*, *e*, *g*, *x*, *y*, *z* are adjustable parameters. *a* is the critical degree of compression; when the explosive is compressed to a certain value, it begins to ignite. In general, the fuel consumption power in ignition and combustion terms is *b* = *c*. The specific parameters of the compBJJ1 explosive are listed in Table 5.

#### 3.2.4. Material Properties of the Shell, Panels, and Liner

In this article, both the shell and front and rear panel materials were chosen as steel_1006, the material exhibits a certain strain rate effect. The Johnson-Cook model shows very good advantages in describing the strain rate effect of metal materials. Therefore, the equation of state was chosen as the shock equation of state incorporating the Johnson Cook strength model as follows:

(14) $$\sigma_{y}=\left(A+B\varepsilon_{p}^{n}\right)\left(1+C\ln{\dot{\varepsilon }}^{*}\right)\left(1+T^{*m}\right)$$

where *σ*_y_ is the yield stress, *A*, *B*, *C*, *n*, *m* are the material parameters, *ε*_p_ is the equivalent plastic strain, ${\dot{\varepsilon }}^{*}$ is the equivalent strain rate, and $T^{*m}$ is the reduced temperature. The liner includes four kinds of materials, copper, floatglass, Lucite, and Plexiglas. The equation of state of copper, Lucite, and Plexiglas is the shock equation of state, while the equation of state of floatglass is the polynomial equation of state and the strength model, the failure model of which is Johnson-Holmquist. 

## 4. Analysis of the Numerical Simulation Results

### 4.1. Comparison of Shaped Jet Formability

**Figure 3:**
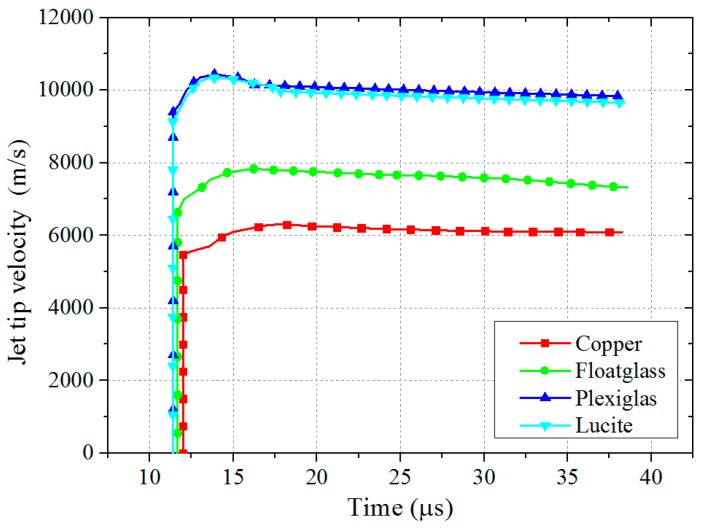
Jet tip velocity corresponding to the four different liner materials.

**Figure 4:**
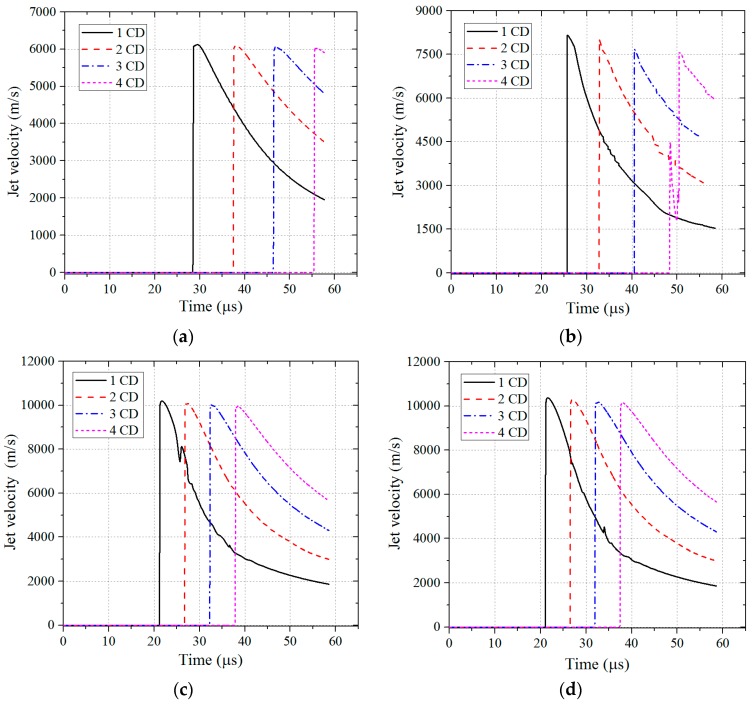
The velocity history corresponding to different stand-offs and different liner materials: (**a**) copper; (**b**) floatglass; (**c**) Lucite; and (**d**) Plexiglas.

When the detonation wave reaches the top of liner, the liner begins to be crushed under the action of detonation pressure and then closes. Subsequently, collision and squeezing occur on the axis, and the shaped jet with high temperature and pressure is formed. The forming states of four different types of shaped jets at *t* = 35 μs are shown in Table 6. In addition, by extracting the historical information of the Gauss points, the jet tip velocity corresponding to the four different liner material types is shown in Figure 3, and the velocity history of four kinds of jets corresponding to different stand-offs (1 CD, 2 CD, 3 CD, 4 CD, where CD is the abbreviation for the charge diameter) were obtained, as shown in Figure 4.

From the shaped jet configuration shown in Table 6, it can be seen that the jet length corresponding to the four different liner materials at the same time is *L*_Plexiglas_ ≈ *L*_Lucite_ > *L*_floatglass_ > *L*_copper_, which indicates that the ductility of Plexiglas and Lucite is closer, while floatglass is worse, and copper is the worst. There exists a temperature rise during the process of shaped jet formation, which mainly comes from two parts: the shockwave and plastic strain. Under normal circumstances, the shaped jet temperature distribution characteristic is as follows: the surface temperature is generally around 400–500 °C, much lower than the center temperature of about 900–1000 °C. Since the melting point of copper is 1083 °C, it does not reach the melting point under the action of the shaped charge. Therefore, the copper shaped jet is not in a melted state, but in a plastic state of high-speed flow, which is different from the general fluid. The Vicar softening temperature of floatglass, Lucite, and Plexiglas are about 720–730 °C, ~113 °C, and ~108 °C, respectively. When the amorphous material is heated to the Vicar softening temperature, the liner material will soften, and then continue to heat up, which will gradually be able to flow, and the higher the temperature, the better the fluidity. From the above analysis based on melting point and softening point, it is obvious that the ductility of three low-density amorphous materials under the action of the shaped charge is superior to that of copper.

In addition, it can be seen from Figure 3 and Figure 4, and Table 6 that the jet tip velocity was *V*_Plexiglas_ ≈ *V*_Lucite_ > *V*_floatglass_ > *V*_copper_, and the corresponding jet mass transfer rate of the four different liner materials also showed *η*_Plexiglas_ ≈ *η*_Lucite_ > *η*_floatglass_ > *η*_copper_. The effective charge at the top of liner is relatively greater, while the effective charge at the bottom of the liner is relatively less, thus the crushing velocity and jet velocity is lower than the former. It can be seen that the jet tip velocity is higher than the tail velocity, namely, there is a velocity gradient, so that the entire jet is continuously elongated until the jet is pulled off. Moreover, the lower the density of the liner material, the more easily it moves forward under the detonation wave, which means that the velocity and mass conversion rate of the low-density shaped jet is higher. 

**Figure 5:**
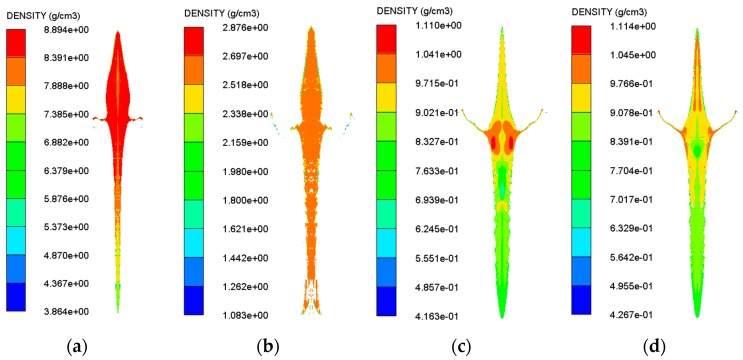
The jet density state at the distance of 3 CD for different liner materials: (**a**) copper; (**b**) floatglass; (**c**) Lucite; and (**d**) Plexiglas.

For most liner materials, when the top of the liner is subjected to detonation waves, the liner begins to be crushed and deformed, which results in a rise in the density of the liner material at this point. However, due to the presence of the velocity gradient, the material is gradually stretched. There will be a coexistence of compression and tensile states, which causes a sharp fluctuation in the jet density, but as the shaped jet is stretched, the density decreases rapidly in the fluctuation. Eventually, when the shaped jet breaks, the material density remains essentially constant. The shaped jet density state at the distance of 3 CD is shown in Figure 5, corresponding to four different types of liner.

There is a certain gradient in the overall density of the shaped jet at the distance of 3 CD as shown in Figure 5. However, the floatglass′s overall jet density is consistent. The density variation of four different jet types is shown in Table 7. Since the jet density has changed during the formation of the shaped jet, $\rho_{j}$ should denote the density at the moment when the shaped jet impacts the target plate instead of the initial density in the *u*-*d* initiation criterion $K=u^{2}d={\left[v/\left(1+\sqrt{\rho_{\mathrm{HE}}/\rho_{j}}\right)\right]}^{2}d$.

### 4.2. Response Analysis of the ERA

**Figure 6:**
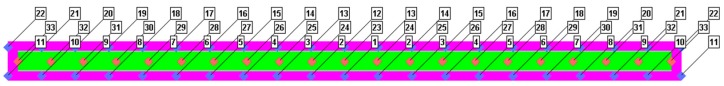
The distribution of Gauss points in ERA.

In the numerical simulation, it is possible to judge whether the sandwich charge reacts according to the particle velocity of panel, the pressure and reaction degree *α* of sandwich charge. Specifically, *α* represents the ratio of the reacted part to the whole part in the explosive, and the range of α is from 0 to 1. If *α* = 0, it indicates that the explosive does not react; 0 < *α* <1, it indicates that the explosive does not react completely; and if *α* = 1, it indicates that the explosive has completely reacted. Therefore, a series of Gauss points were set inside the ERA to observe the particle velocity of panel, the pressure and reaction degree *α* of sandwich charge, as shown in Figure 6.

#### 4.2.1. Reaction Degree *α* of the Sandwiched Charge

**Figure 7:**
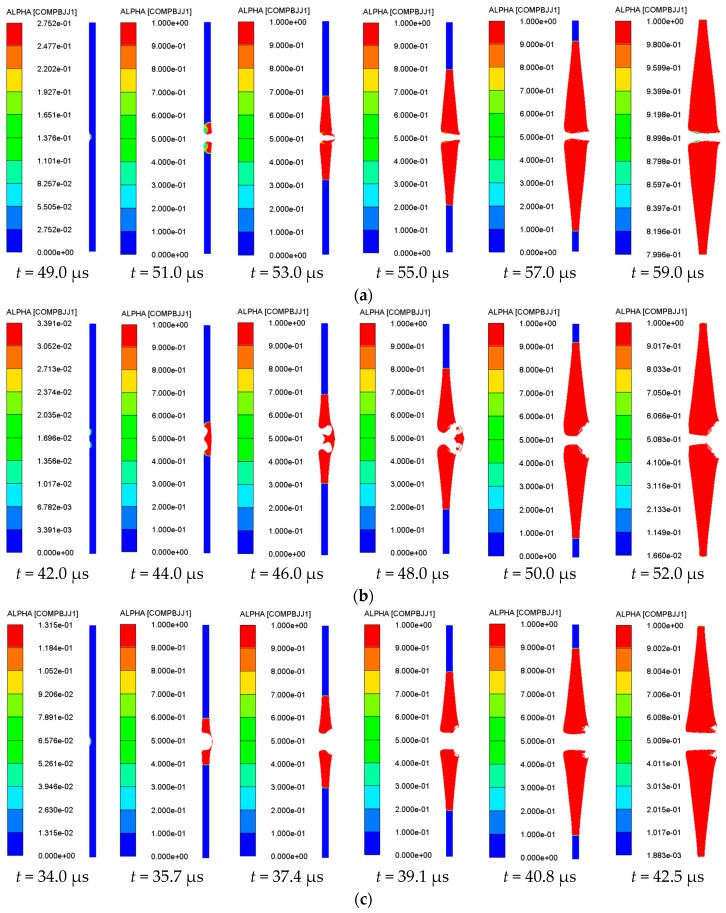

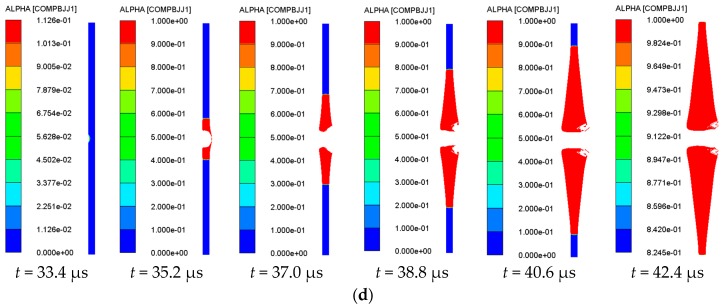
The reaction degree contour of the sandwiched charge corresponding to different jet types: (**a**) copper; (**b**) floatglass; (**c**) Lucite; and (**d**) Plexiglas.

In order to study the reaction process of sandwiched charge under the impact of shaped jet, the reaction degree of sandwich charge corresponding to four different types of jets at different times was studied. There are six different moments for each set of working conditions, and the entire time range covers the formation of hot spots to the complete detonation of the sandwiched charge. The reaction degree contour of the sandwiched charge corresponding to different types of jet is shown in Figure 7.

**Figure 8:**
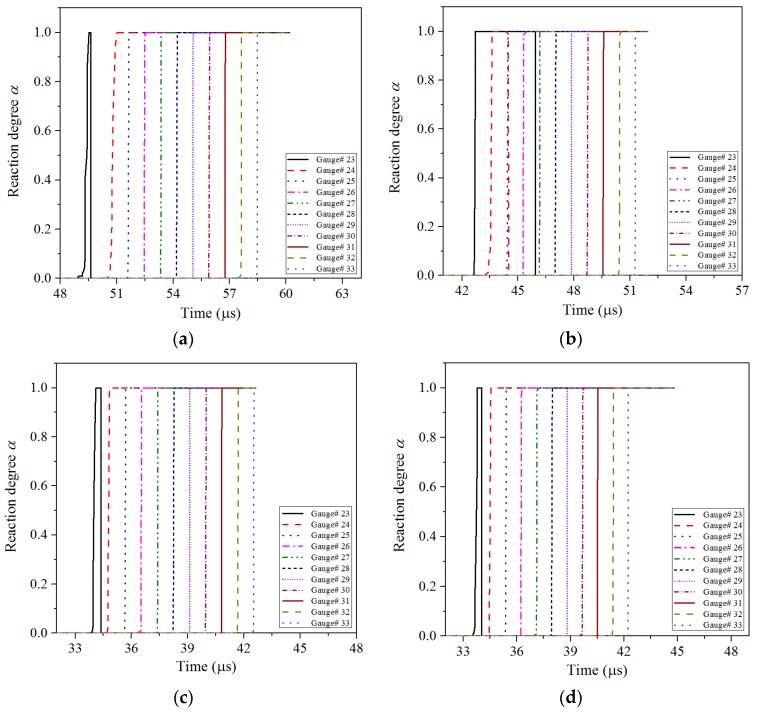
The reaction degree *α* of Gauss points in the middle of the sandwich charge for different liner materials: (**a**) copper; (**b**) floatglass; (**c**) Lucite; and (**d**) Plexiglas.

It can be seen from Figure 7, when the impact intensity reaches a certain value under the impact of shaped jet, the local high temperature area is formed, which is called a hot spot. With further penetration of the shaped jet, the pressure, density, and temperature at the wave front increase sharply, which breaks the thermal balance around the hot spot regions inside the charge. When the hot spot temperature is higher than the thermal decomposition temperature of the explosive, the explosive decomposes and generates energy, with heat generating and expanding at the center of the hot spot to the surrounding explosive, which will eventually lead to the hot spot growth. When the explosive breaks down to a certain extent, the hot spots will begin to connect, and the strong reaction formed by the hot spots will lead the explosive into low-speed detonation, and then grow into a stable detonation. As a result, the reaction degree *α* reaches 1 rapidly, it spreads to the surrounding diffusion at a certain rate, and then extends to the entire area of the sandwiched charge. In addition, by observing the reaction degree *α* of Gauss points in the middle of the sandwich charge, it can be seen from Figure 8 that the sandwich charge has achieved the complete detonation process.

According to the kinetic energy theorem, the kinetic energy of the jet is proportional to the square of the velocity, so the larger the velocity is, the greater the kinetic energy is. The density of three amorphous materials is lower, the jet velocity obtained is higher, so its energy is higher, and it is more likely to initiate the sandwich charge for the three amorphous materials. The time of four shaped jets from impacting the sandwich charge to fully detonating the sandwich charge is *t*_floatglass_ = 9.5 μs, *t*_Lucite_ = 9.0 μs, *t*_Plexiglas_ = 8.9 μs, *t*_copper_ = 10.7 μs, respectively.

Based on the simulation results of the tip velocity, tip diameter and density of shaped jet, it is possible to judge whether the jet can initiate the sandwich charge from the theoretical point of view according to the *u*-*d* initiation criterion $K=u^{2}d={\left[v/\left(1+\sqrt{\rho_{\mathrm{HE}}/\rho_{j}}\right)\right]}^{2}d$. For the compBJJ1 explosive, its critical initiation threshold is *K* = 23 mm^3^/μs^2^, and the calculation results are shown in Table 8.

It can be seen from Table 8 that the values of *u*^2^*d* corresponding to the four different material liners are greater than 23 mm^3^/μs^2^, which shows that the sandwich charge can be reliably initiated from the theoretical point of view. In addition, the numerical simulation results in Figure 8 show that the four kinds of jets all initiate the sandwich charge, which indicates that the numerical simulation method in this article is reliable.

#### 4.2.2. Detonation Pressure of the Sandwiched Charge

**Figure 9:**
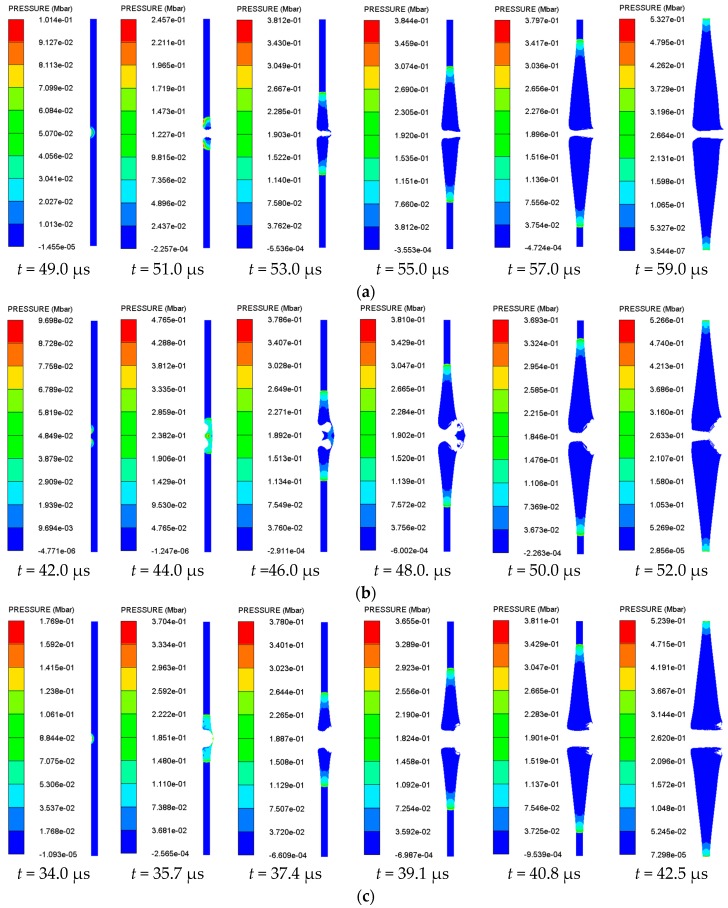

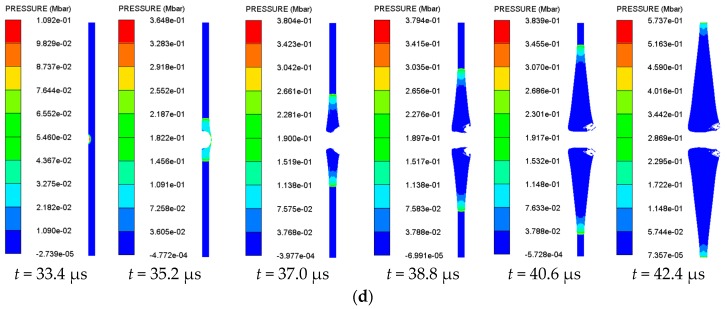
The pressure contour of the sandwich charge corresponding to different jet types: (**a**) copper; (**b**) floatglass; (**c**) Lucite; and (**d**) Plexiglas.

In addition, the detonation pressure can also be used to determine whether the sandwiched charge is initiated. The pressure contours at the same time as those in Figure 7 were extracted, as shown in Figure 9.

**Figure 10:**
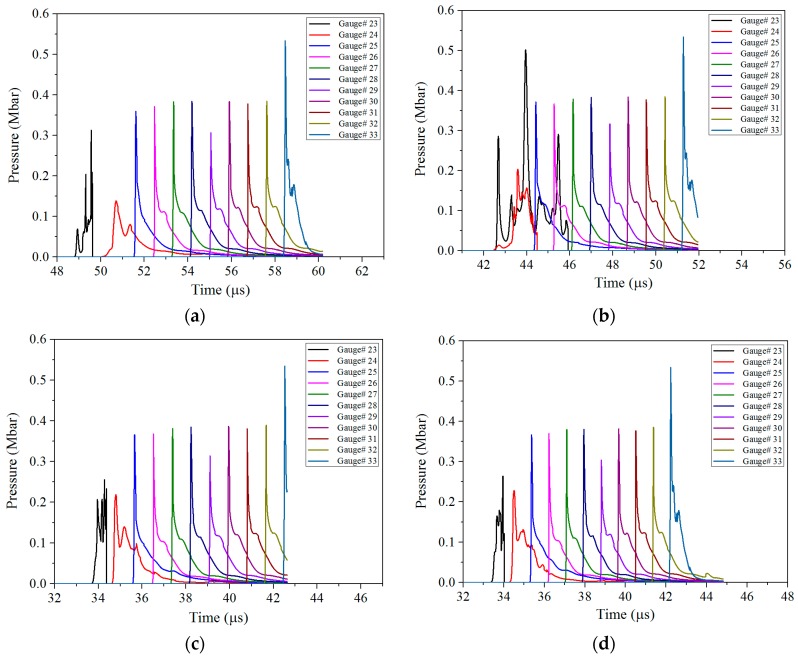
Pressure history curves of Gauss points in the sandwich charge for different liner materials: (**a**) copper; (**b**) floatglass; (**c**) Lucite; and (**d**) Plexiglas.

The formation and growth into stable detonation waves of hot spots in the sandwiched are clearly shown in Figure 9. Since the tip of the floatglass shaped jet is hollow, it is equivalent to two shock waves propagated into the sandwich charge in the two-dimensional plane. The two shock waves must be superimposed on the axis position, and the pressure state corresponding to *t* = 44.0 μs in Figure 9b can be well illustrated at this point. In order to analyze the change of the detonation pressure in the sandwich charge more intuitively, the pressure history curve shown in Figure 10 was obtained by extracting the pressure history of the Gauss points in the sandwich charge.

It can be seen from Figure 9 and Figure 10 that the sandwiched charge is initiated by the shaped jet when the impact pressure reaches the initiation pressure threshold. In addition, the pressure within the charge increases instantaneously and is close to the detonation pressure. With further penetration of the shaped jet, the stable detonation wave formed in charge is transmitted to both ends at a steady speed.

#### 4.2.3. Particle Velocity of the Front and Rear Panels

**Figure 11:**
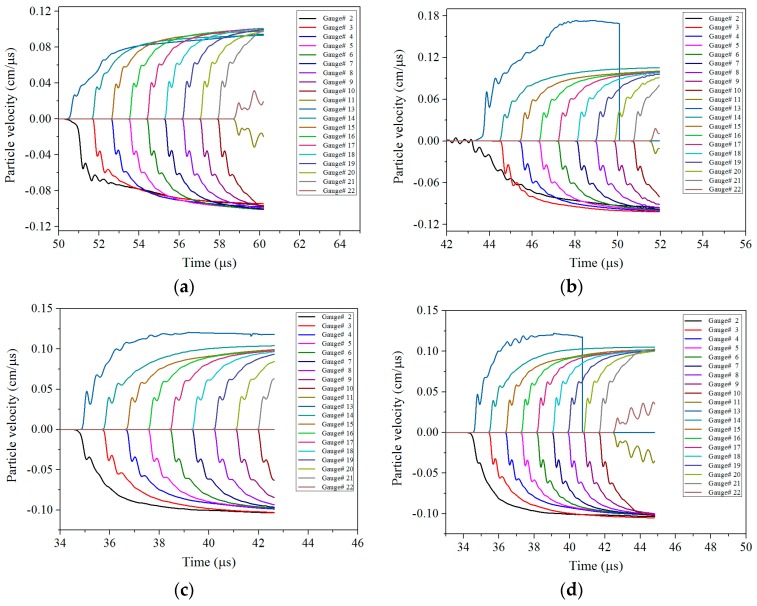
The particle velocity of the Gauss points in the front and rear panels for different liners: (**a**) Copper; (**b**) Floatglass; (**c**) Lucite; and (**d**) Plexiglas.

For the ERA, if the sandwiched charge is initiated, the particle velocities of the front and rear panels will increase dramatically. On the contrary, if there is no explosion in the sandwiched charge, there is almost no change in the particle velocity of the front and rear panels. Therefore, in addition to the above criteria which based on the reaction degree and pressure, the particle velocity of the front and rear panels can also be used as a criterion. To this end, the particle velocity of the Gauss points in the front and rear panels was extracted, as shown in Figure 11.

The Gauss point distribution of the front and rear panels is shown in Figure 6, where Gauss points 1–11 are distributed in the front panel, and Gauss points 12–22 are distributed in the rear panel. However, since the Gaussian points 1 and 12 are located on the axis, they are rapidly eroded when the shaped jet passes though. Thus, the particle velocity history of the Gauss points 2–11 of the front panel and the Gauss points 13–22 on the rear panel were extracted only in this article.

It can be seen from Figure 11 that the maximum velocity of the particle velocity of the front and rear panels corresponding to the four different working conditions is almost 1000 m/s. The particle velocity of the front panel is slightly smaller than the particle velocity of the rear panel, but it exhibits a good symmetry, which is consistent with the actual situation. Since the front and rear panel obtained a certain forward movement velocity under the impact of shaped jet, when the sandwich charge is initiated, the front panel obtains a larger negative velocity and the rear panel obtains a larger positive velocity, which makes the resultant velocity of front panel relatively small. In addition, the closer to the ends of the ERA, the difference will become smaller. In Figure 11b,d, the velocity of Gauss point 2 suddenly appears unloading, because the mesh near the point experiences a large distortion and the point is deleted.

## 5. Conclusions

Using a low-density material as the liner of the shaped charge pre-warhead to destroy the ERA is proposed in this article, and its effectiveness is proved by the numerical simulation that it is feasible to perforate and initiate the ERA through the low-density shaped jet. According to the *u*-*d* initiation criterion, the *u*^2^*d* of the three low-density shaped jets is greater than the critical value of 23 mm^3^/μs^2^. It can be concluded that the low-density shaped jet has the following advantages compared with the conventional metal (copper) shaped jet:

(1) The mass of three kinds of low-density liners are smaller than that of copper liner under the same volume condition.
(2) When the shaped jet just contacts the target plate, the tip diameters of copper, floatglass, Lucite, and Plexiglas jets are 2.6 mm, 5.2 mm, 6.0 mm, and 6.0 mm, corresponding to tip velocities of 6042 m/s, 7464 m/s, 9785 m/s, and 9849 m/s, respectively, so the tip diameter and velocity of the three kinds of low-density shaped jets is greater than copper. In addition, the respective shaped jet length of copper, floatglass, Lucite, and Plexiglas is 84 mm, 98 mm, 137 mm, and 141 mm at *t* = 35 μs, which indicates that the ductility of the three low-density materials is also better than copper in the same structure of the shaped charge.
(3) The time of three kinds of low-density shaped jets from impacting the sandwich charge to complete detonation (*t*_floatglass_ = 9.5 μs, *t*_Lucite_ = 9.0 μs, *t*_Plexiglas_ = 8.9 μs) is also shorter than that of copper shaped jet (*t*_copper_ = 10.7 μs).

The above advantages make the low-density shaped jet a great application prospect. In the three kinds of low-density materials, the density of Lucite and Plexiglas are close to the minimum. At the same time, the floatglass shaped jet appears to be hollow in the jet tip during the stretching process; in other words, the floatglass has no good formablity compared to the other two low-density shaped jets. Therefore, the Lucite and Plexiglas can be considered as the two best low-density materials in this article. In summary, this study provides a certain value on the possible application of low-density liner materials in the shaped charge-shaped charge type tandem warhead, and it would be a plausible and sustainable direction for further research.

## Figures and Tables

**Table 1:** Geometrical parameters of the entire model.

Shell Length	Shell Thickness	Shell Diameter	Cone Bottom Diameter	Liner Thickness	Front Panel Thickness	Sandwich Charge Thickness	Rear Panel Thickness
135 mm	3 mm	60 mm	54 mm	3 mm	2 mm	4 mm	2 mm

**Table 2:** The specific numbers and coordinates of Gauss points.

Part	Gauss Point Number	Gauss Point Coordinate		INTERVAL		Gauss Point Type
		x/mm	y/mm	∆x/mm	∆y/mm	
Front panel	1–11	29.8	0–70	-	7	Moving
Rear panel	12–22	30.4	0–70	-	7	Moving
Sandwich charge	23–33	30.1	0–68	-	6.8	Fixed
Air	34–153	93–450	0	3	-	Fixed

**Table 3:** Material properties of air.

Material	*ρ* (kg/m^3^)	*γ*	*C_p_* (kJ/kg∙K)	*C_v_* (kJ/kg∙K)	*T* (K)	*E*_0_ (kJ·kg^−1^)
air	1.225	1.4	1.005	0.718	288.2	2.068 × 10^5^

**Table 4:** The JWL parameters and C-J parameters of the compB explosive.

Material	*A* (Mbar)	*B* (Mbar)	*C* (Mbar)	*R*_1_	*R*_2_	*ω*	*ρ*_0_ (g/cm^3^)	*P*_CJ_ (Gpa)	*D* (m/s)
CompB	5.242	0.07678	0.01082	4.20	1.10	0.34	1.717	29.5	7980

**Table 5:** Parameters of Lee-Tarver ignition growth model for the compBJJ1 explosive.

JWL	*A* (Mbar)	*B* (Mbar)	*R*_1_	*R*_2_	*C*_v_ (Mbar/K)	*G* (Mbar)	*σ*_s_ (Mbar)	*ω*
Unreacted	778.1	−0.0503	11.3	1.13	2.487 × 10^−5^	0.0354	0.002	0.894
Product	5.242	0.0768	4.2	1.1	1.0 × 10^−5^	-	-	0.34
Reaction Rates	*a*	*b*	*c*	*d*	*e*	*g*	*I* (μs^−1^)	FG_1max_
	0.01	0.222	0.222	0.667	0.0	0.0	44	1.0
	FG_2min_	Fig_max_	*x*	*y*	*z*	*G*_1_ (Mbar^−2^·μs^−1^)	*G*_2_ (Mbar^−2^·μs^−1^)	
	1.0	0.3	4	2	0.0	414	0.0	

**Table 6:** Configuration and performance parameters of shaped jets.

Material	Shaped Jet Configuration	Jet Tip Velocity (m/s)	Jet Tail Velocity (m/s)	Jet Length (mm)	Pestle Length (mm)
Copper	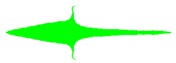	6088	138	84	56
Floatglass	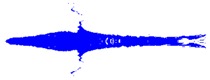	7421	953	98	58
Lucite	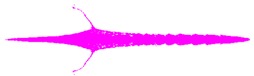	9715	667	137	77
Plexiglas	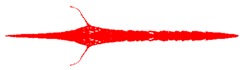	9871	632	141	78

**Table 7:** The density variation of four different shaped jet types.

Material	Initial Density *ρ*_initial_ (g/cm^3^)	Current Density *ρ*_current_ (g/cm^3^)	Variable Quantity of Density Δ*ρ* (%)
Copper	8.93	7.02	−21.4%
Floatglass	2.53	2.53	0%
Lucite	1.181	0.763	−35.4%
Plexiglas	1.186	0.776	−34.6%

**Table 8:** The calculation results of the *u*-*d* initiation criterion.

Material	Jet Tip Velocity *v*_t_ (m/s)	Jet Tip Diameter *d*_t_ (mm)	Density *ρ* (g/cm^3^)	Initiation Criterion *u*^2^*d* (mm^3^/μs^2^)	Detonation Situation
Copper	6042	2.6	7.02	42.49	Yes
Floatglass	7464	5.2	2.53	87.09	Yes
Lucite	9785	6.0	0.763	91.91	Yes
Plexiglas	9849	6.0	0.776	94.06	Yes
